# Ablation of typical atrial flutter using mini electrode measurements for maximum voltage‐guided ablation: A randomized, controlled trial

**DOI:** 10.1002/joa3.12665

**Published:** 2021-12-09

**Authors:** Matthew K. Rowe, Andrew Claughton, Jason Davis, Lauren Yee, Gerald C. Kaye, Kieran Dauber, John Hill, Paul A. Gould

**Affiliations:** ^1^ Department of Cardiology Princess Alexandra Hospital Brisbane Queensland Australia; ^2^ The University of Queensland Brisbane Queensland Australia

**Keywords:** ablation, atrial flutter, cavotricuspid isthmus, MiFi, mini electrode

## Abstract

**Background:**

Novel ablation catheters with mini electrode (ME) sensing have become available but their utility is unclear. We investigated whether ablation of the cavotricuspid isthmus (CTI) for atrial flutter (AFL) would be improved using ME signals.

**Methods:**

Sixty‐one patients (76% male, 63 ± 10 years) with CTI‐dependent AFL underwent ablation using a maximum voltage‐guided approach, randomized to either standard 8 mm non‐irrigated catheter with bipolar signals or IntellaTip MiFi catheter using ME signals alone.

**Results:**

Acute bidirectional block was achieved in 97%. Mean follow‐up was 16.7 ± 10 months. The median number of ablation lesions was 13 in both groups (range 3–62 vs. 1–43, *p* = .85). No significant differences were observed in AFL recurrences (17% vs. 11%, *p* = .7), median procedure durations (97 min [interquartile range (IQR), 71–121] vs. 87 min [IQR, 72–107], *p* = .55) or fluoroscopy times (31 min [IQR, 21–52] vs. 38 min [IQR, 25–70], *p* = .56). Amplitudes of ME signals were on average 160% greater than blinded bipolar signals. In 23.7% of lesions where bipolar signals were difficult to interpret, 13.6% showed a clear ME signal.

**Conclusions:**

There was no difference in the effectiveness of CTI ablation guided by ME signals, compared with using bipolar signals from a standard 8 mm ablation catheter. While ME signal amplitudes were larger and sometimes present when the bipolar signal was unclear, this did not improve procedural characteristics or outcomes. The results suggest future research should focus on lesion integrity rather than signal sensing.

## Graphical abstract

In a randomized controlled trial during catheter ablation of typical atrial flutter, the use of mini electrode (ME) signals on the MiFi catheter was compared to standard of care bipolar signals on an 8‐mm catheter. While ME signal amplitudes were larger and sometimes present when bipolar signal was unclear, there was no difference in procedural characteristics or outcomes between the two groups.

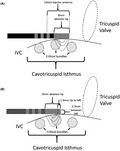

## 1 INTRODUCTION

Catheter ablation is a class I indication for symptomatic cavotricuspid isthmus (CTI) dependent atrial flutter (AFL).^1^ Recurrence, however, following acutely successful CTI ablation remains an issue, and rates are approximately 10%, even when the bidirectional block has been achieved.^2^, ^3^ Repeat studies post‐CTI ablation have demonstrated up to 30% of patients have a recurrence of conduction across the CTI.^4^ Recurrence may be because of procedural characteristics such as failure to achieve a transmural lesion or the inability to detect residual conduction through areas of ablated tissue. Previous pathological studies have shown that the CTI is non‐uniform and is comprised of functionally discrete muscle bundles surrounded by connective tissue which can be targeted for ablation to achieve bidirectional CTI block.^5^, ^6^

**FIGURE 1:**
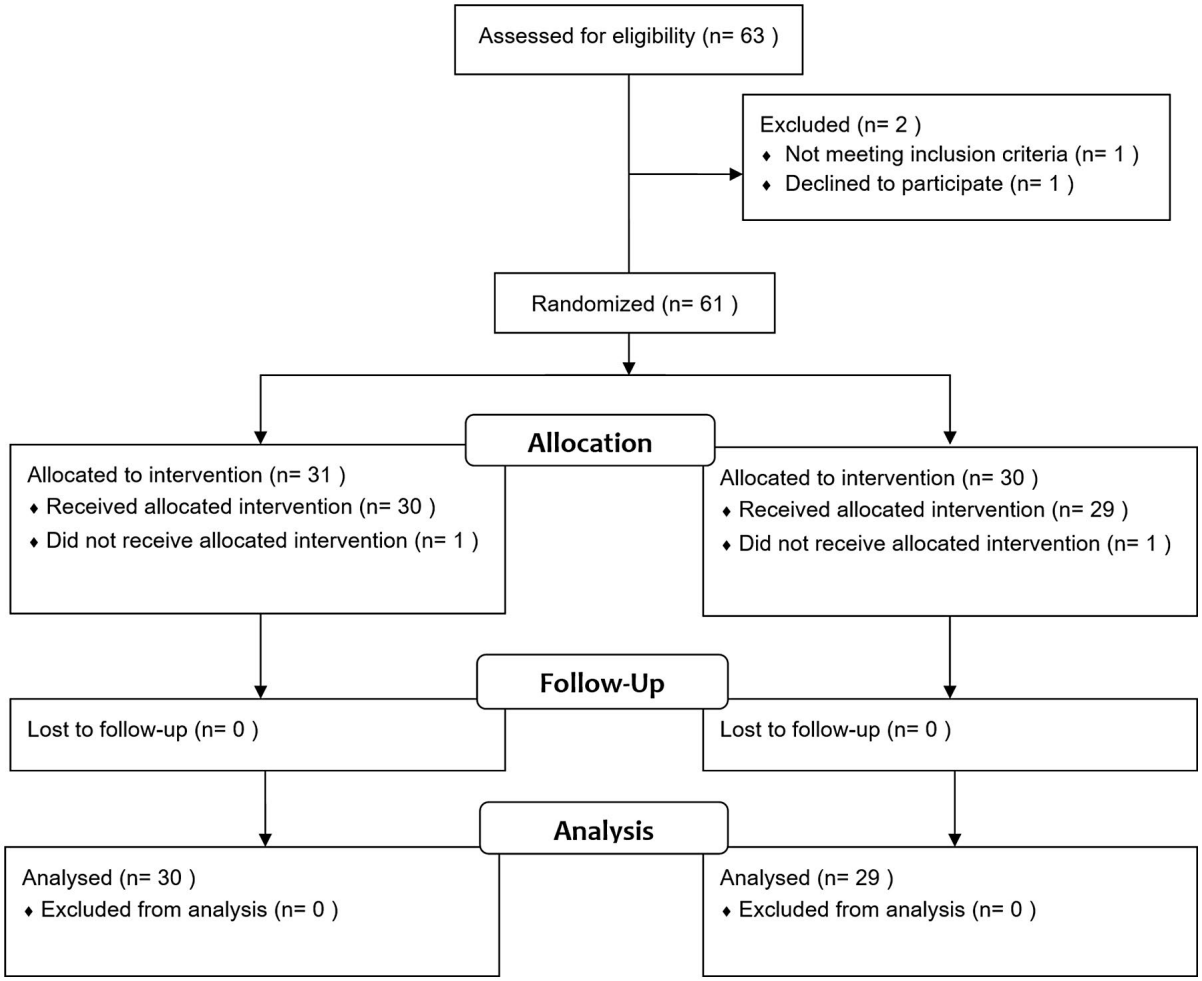
CONSORT diagram showing participant flow through each phase of the trial

Traditional markers of effective ablation include electrogram (EGM) signal diminution, impedance change and ablation duration. We have previously demonstrated that incorporating measurement of contact force (CF) and force‐time integral (FTI) may prevent AFL recurrence post‐CTI ablation.^2^ To improve signal sensing, the IntellaTip MiFi XP catheter (Boston Scientific) contains three mini electrodes (ME) only 1.3 mm from the catheter tip (Figure 1). This enables the measurement of a bipolar signal from any two of three radially arranged, 0.8 mm wide electrodes. The comparable bipole distance is 10 mm in a conventional 8 mm tip catheter. This smaller 1.3 mm field should enable more accurate localization of the anatomical bundles of tissue conducting electrical signal across the CTI to target for ablation and avoid ablation of “far‐field” signals. Additionally, ME signals have previously been demonstrated to be helpful in localizing gaps across linear ablation lesions.^7^

The aim of this study was to assess whether CTI ablation using ME signals would be superior to using standard bipolar signals by enabling critical regions to be identified and reducing the amount of ablation required; therefore, reducing procedure and fluoroscopy times, and whether a difference in recurrence rates could be seen.

## 2 METHODS

Consecutive patients with ECG documented AFL referred for CTI ablation were enrolled at 2 sites: Princess Alexandra Hospital and St Andrew's War Memorial Hospital in Brisbane, Australia. The study was investigator‐initiated and the study protocol was approved by the research ethics board for research involving human subjects at each participating center and complies with the declaration of Helsinki. Each operator in the study was an experienced electrophysiologist with >500 prior ablation procedures. No patients declined to participate in the study.

### 2.1 Exclusion criteria

Patients were excluded from the study if they had any of the following: Age <18 years, inability to consent, congenital or significant structural heart disease (defined as LVEF <45%), pregnancy, severe renal impairment (eGFR <20 ml/min), repeat procedure, or in atrial fibrillation at the time of the study.

### 2.2 Randomization and blinding

After informed consent, patients were assigned, via computer‐generated block randomization, to either the control or intervention group in a 1:1 fashion. The patient was blinded to the assigned treatment. In the intervention arm, the operator was blinded to the traditional bipolar signals and relied only on the ME signals during ablation.

### 2.3 Ablation catheter

In the control cohort, an 8‐mm non‐irrigated catheter (Celsius DS, Biosense Webster) with 1–6–2‐mm electrode spacing was used. In the intervention group, the MiFi catheter, an 8‐mm tip non‐irrigated catheter with 3 ME sensors 1.3 mm from the catheter tip was used. Power was set initially to 40 W, which could be increased at the operator's discretion, and the temperature limited to 65°C with a specified ablation time of 60 s per lesion or, if not, at least 50% diminution of EGM signal.

### 2.4 Ablation procedure

Patients underwent the procedure in a fasted and consciously sedated state. Right femoral vein access was obtained under local anesthesia and three venous sheaths sited for the ablation catheter, coronary sinus catheter (6F IBI, Abbott Laboratories); and a 20‐pole mapping catheter (7F Livewire, Abbott Laboratories) in the right atrium. The use of long sheaths was at the discretion of the operator. For patients in atrial flutter, involvement of the CTI in the circuit was confirmed via overdrive pacing from the CTI region and measurement of the post‐pacing interval (PPI).^8^ Bipolar intra‐cardiac EGMs (measured from the ablation catheter tip electrode to the distal electrode with 2 mm inter‐electrode spacing) and 12‐lead surface ECG were recorded simultaneously on a computerized digital amplifier system. Mini electrode signals were measured from the three tip electrodes generating 3 vectors: poles 1–2, 2–3, and 3–1. The EGM filtering was set between 30 and 500 Hz.

The ablation approach employed was a maximal voltage‐guided (MVG), point‐by‐point technique, which has been shown previously to be more effective than the “pull back” method.^9^ The site of ablation was determined by mapping along the CTI and targeting the site with the largest EGM. In the MiFi catheter cohort, the operator was blinded to the standard bipolar signals and only the maximum voltages from the local ME were used to guide ablation. In the control cohort, ablation was guided by standard bipolar signals. The endpoint of the procedure was bidirectional block across the CTI, which was defined as an increase in the trans‐isthmus conduction time of >50% and reversal of the right atrial activation sequence seen on the duodecapolar catheter when pacing from either side of the ablation line.^8^ A waiting period of 30 min was observed with bidirectional block re‐confirmed every 5 min.

### 2.5 Outcomes

The primary endpoint was the number of ablation lesions required to achieve acute bidirectional block across the CTI. Secondary endpoints were procedure duration, fluoroscopy time, acute procedural success, and recurrence of typical flutter, documented on ECG or Holter monitor, at 12‐month follow‐up. Safety endpoints were peri‐ or post‐operative events with major events defined as death, myocardial infarction, stroke, pulmonary embolus, pericardial tamponade/effusion, major bleeding, or need for a permanent pacemaker. All other complications were considered minor.

### 2.6 Data collection

For each ablation lesion the following data were recorded: The EGM maximum amplitude (mV) before and at the end of ablation, measured at the 2 mm distance distal electrode, above and below the baseline, the EGM of the 3 ME signals in the MiFi group, impedance (Ohms) before and at end of ablation and the ablation duration in seconds. The absolute and percentage change in EGM amplitude and impedance (“impedance drop”) were then calculated. Bipolar electrograms were also recorded in the MiFi group although the operator was blinded to them. Procedure times and fluoroscopy times were collected, except in the cases of patients undergoing concurrent pulmonary vein isolation (*n* = 6) since only the total times were available for these cases.

### 2.7 Clinical follow‐up

Patients were assessed immediately post‐procedure, and at 3 and 12 months with electrocardiograph and 24‐h Holter monitoring. Arrhythmic recurrences were documented both clinically and electrocardiographically to rule out non‐flutter arrhythmias. Recurrence was defined as ECG documented, CTI dependent AFL following a successful procedure. All patients in the study were followed for a minimum of 3 months to be included in the final analysis.

### 2.8 Power calculation and statistical analysis

A reduction in the number of ablation lesions required of 20% was considered the minimum clinically relevant effect. Based on a previous study where the mean number of ablations was 23 ± 6, the sample size required to give 80% power to detect a significant difference with an alpha value of 0.05 was 46 patients. A target enrolment of 60 patients allowed for loss to follow‐up or smaller than expected effect size.

Data are presented as mean ± standard deviation if normally distributed and as a median and interquartile range [25th and 75th percentiles] if non‐normally distributed unless otherwise stated. Statistical analysis was performed using SPSS statistical software (IBM). Within‐group data were compared using a paired *t*‐test and between‐group data were compared using an unpaired *t*‐test for normally distributed data. Non‐normally distributed unpaired data were analyzed with a Mann–Whitney test and paired non‐normally distributed data were analyzed with a Wilcoxon signed‐rank test. Nominal or categorical data were compared using a Chi‐squared test or Fisher's Exact Test where appropriate. A probability value of <.05 was considered statistically significant. All ablation lesions were included in the final analysis on an intention to treat basis regardless of whether targets were met or exceeded.

## 3 RESULTS

**FIGURE 2:**
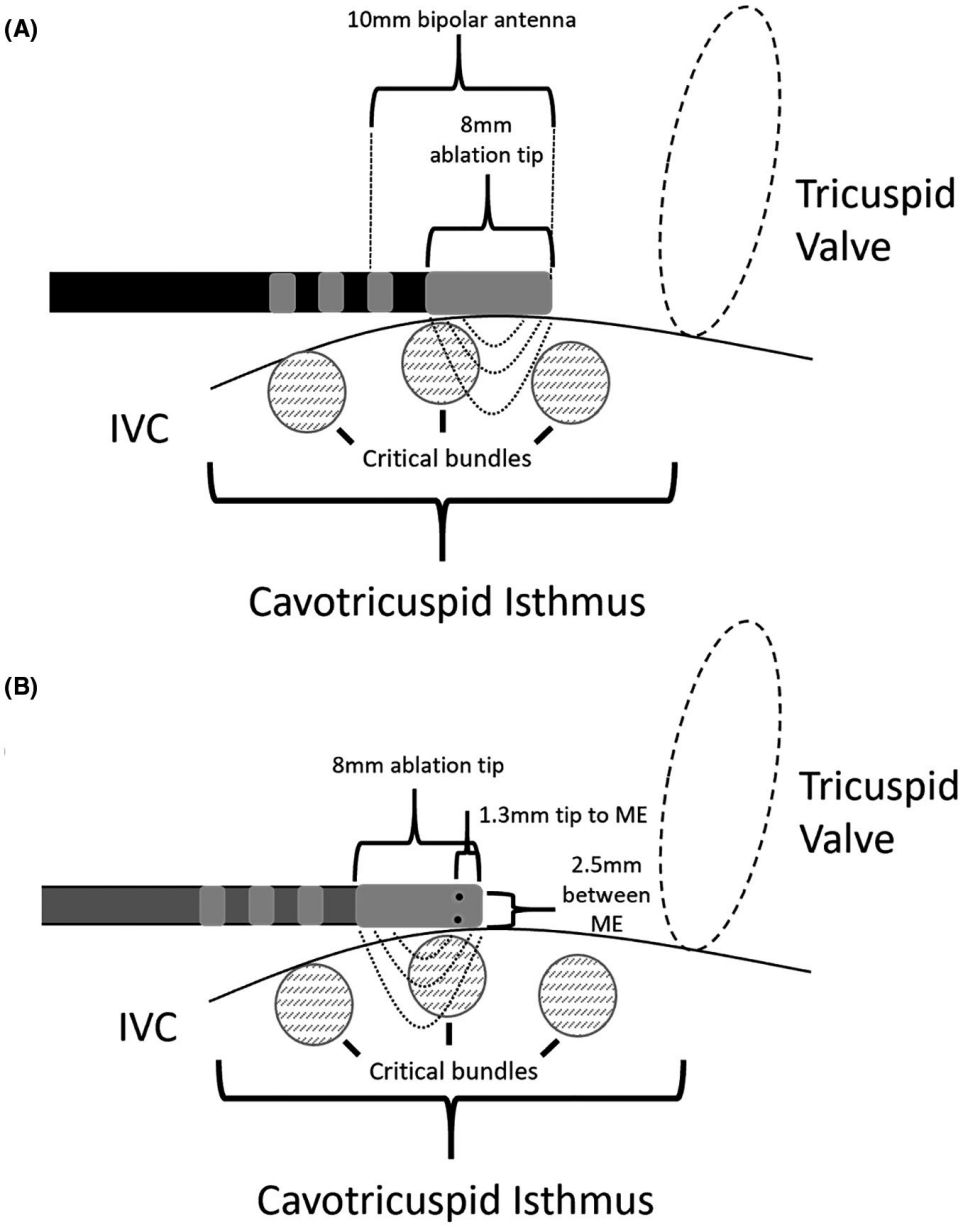
Representation of the recording area and ablation surface of a standard ablation catheter (A) and the MiFi catheter (B) positioned along the cavotricuspid isthmus. IVC, inferior vena cava; ME, mini electrode

Between February 2017 and February 2020, 61 patients were enrolled and randomized (Figure 2). The baseline characteristics of the groups were evenly matched, as shown in Table 1. Acute procedural success, with bidirectional block, was achieved in 97% of patients in both groups. The mean duration of follow‐up was 16.7 ± 10 months.

**TABLE 1:** Baseline patient demographics

	Control (*n* = 30)	MiFi (*n* = 29)	*p*‐value
Age (years)	64 ± 10	62 ± 9	.4
Males, *n* (%)	21 (70)	24 (83)	.2
Ejection fraction (%)	57 ± 8	55 ± 8	.16
Weight (kg)	98 ± 23	101 ± 23	.88
History of atrial fibrillation	9 (30)	14 (48)	.1
CHADS2‐Vasc score	1.6 ± 0.9	1.4 ± 1.1	.62
Atrial flutter at procedure	11 (37)	12 (41)	.92

Procedural characteristics are shown in Table 2. Comparing control and MiFi groups there were no significant differences observed in the primary endpoint of median number of ablation lesions (13 (range 3–62) vs. 13 (range 1–43), *p* = .86), or secondary endpoints of procedure duration (97 [71–121] vs. 87 [72–107] min) or fluoroscopy time (31 [21–52] vs. 38 [25–70] min). There was no difference in recurrence of documented AFL after successful CTI ablation, which occurred in 5 (17%) of the control arm and 3 (11%) of the MiFi arm (*p* = .7). Atrial fibrillation was observed in 4 (14%) and 9 (30%) of patients, respectively, during follow‐up, including 3 new instances, without recurrent AFL, in the MiFi group. The mean ablation duration per lesion was 54 ± 11 versus 51 ± 15 s (*p* = .67). There was no difference between groups in impedance drops (9 [6–12] vs. 8 [6–10] ohms), a traditional marker of effective ablation. Analysis of cases with and without recurrence of AFL did not reveal any meaningful differences in EGM amplitudes or other ablation parameters.

**TABLE 2:** Procedural outcomes

	Control (*n* = 30)	MiFi (*n* = 29)	*p*‐value
Procedural success, *n* (%)	29 (97)	28 (97)	
Procedure duration (min)	97 [71–121]	87 [72–107]	.55
Fluoroscopy time (min)	31 [21–52]	38 [25–70]	.56
Ablation lesions, *n* (range)	13 (3–62)	13 (1–43)	.86
Impedance drop (Ohms)	9 [6–12]	8 [6–10]	.06
Bipolar signal start (mV)	0.3 [0.2–0.6]	0.39 [0.2–0.6]	.48
Bipolar signal end (mV)	0.14 [0.1–0.2]	0.18 [0.1–0.3]	<.01
Bipolar signal change (mV)	−0.14 [0–0.4]	−0.14 [0–0.3]	<.01
Bipolar signal decrease (%)	−57 [29–73]	−45 [15–68]	<.01
ME signal start (mV)		0.54 [0.3–1.1]	<.01^a^
ME signal drop (mV)		0.35 [0.1–0.8]	<.01^a^
ME signal decrease (%)		79 [59–89]	<.01^a^
Follow‐up, months	18 [12–24]	19 [13–28]	
Recurrence, *n* (%)	5 (17)	3 (11)	.7

Abbreviation: ME, mini electrode.

^a^ Compared to bipolar voltage.

**FIGURE 3:**
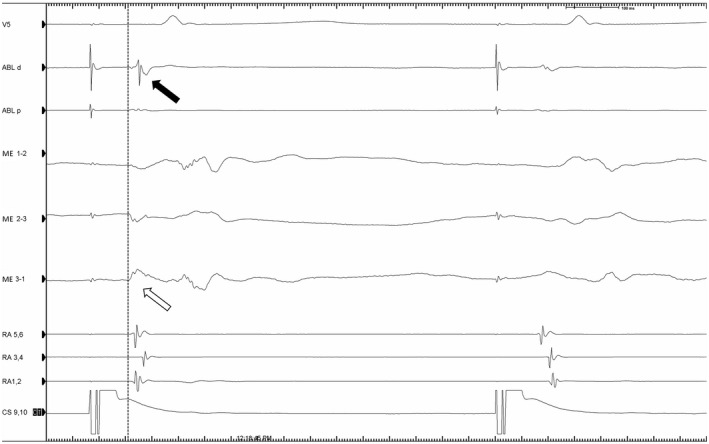
Comparison of ME signal (white arrow) compared to bipolar (black arrow) during ablation which achieved bidirectional CTI block. ME signal is present earlier than bipolar signal. Block is evidenced by the change in RA activation and increase in conduction time to the lateral RA 1,2. ABL, ablation bipolar; CS, coronary sinus; ME, mini electrode; RA, right atrial duodecapolar

**FIGURE 4:**
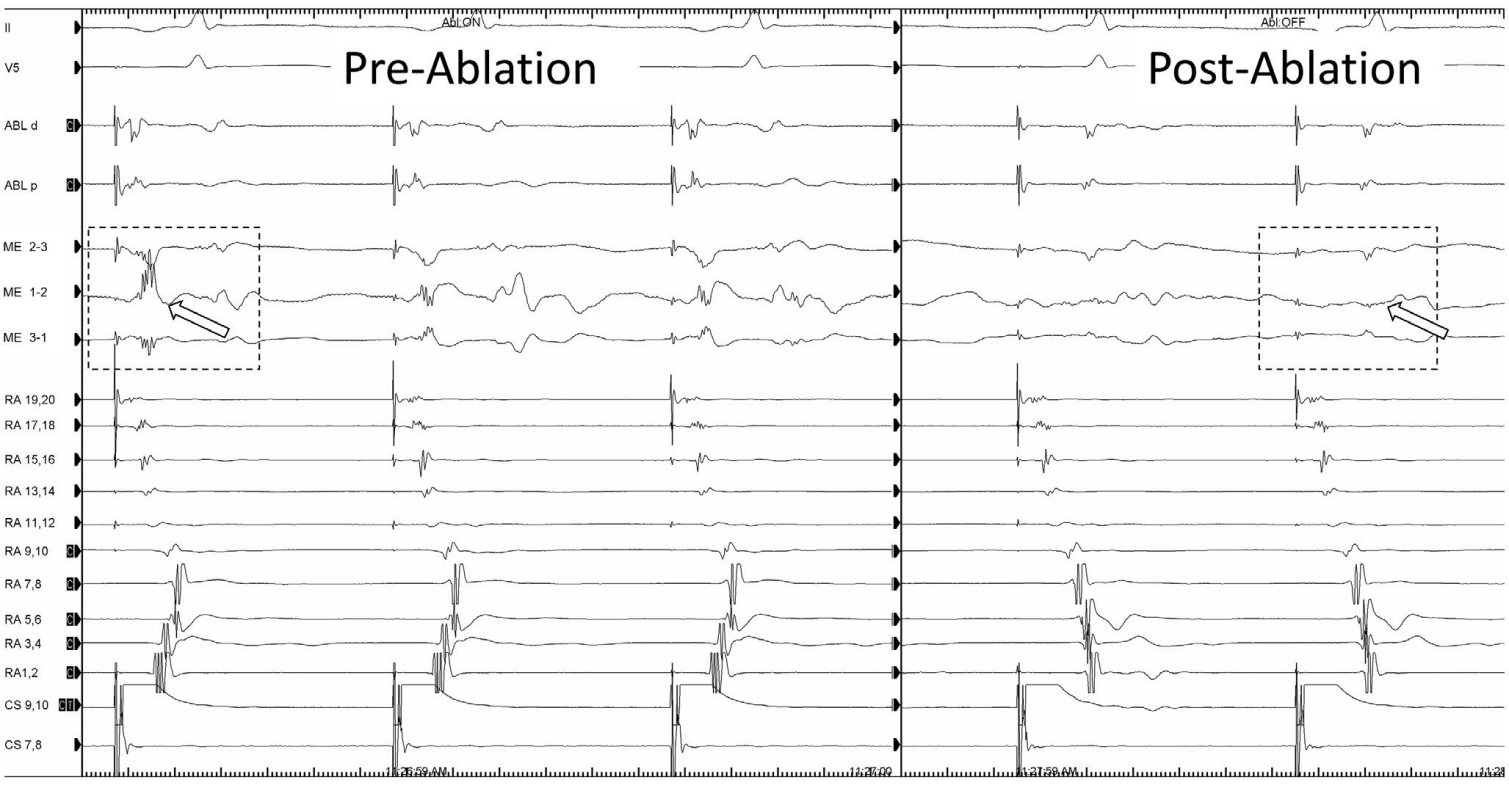
An example of ME signal (white arrows) seen immediately before and after achieving bidirectional CTI block. The MiFi signal is much larger and sits between the septal and lateral signals seen on the DD catheter. Note the rapid reduction in signal amplitude as ablation begins and the near‐complete absence of signal post‐ablation. ABL, ablation bipolar; CS, coronary sinus; ME, mini electrode; RA, right atrial duodecapolar

To evaluate the potential significance of the ME signals, the blinded bipolar signals measured simultaneously from the MiFi catheter were reviewed for each of the 526 total lesions. The frequency of lesions where the ME signal amplitude was 200% greater than bipolar amplitude was calculated and occurred in 30.0% of lesions. Bipolar signals were judged difficult to interpret (<0.2 mV) in 23.7% of lesions but a ME signal was interpretable (>0.5 mV) in 13.6% of these lesions (Figure 3). Conversely, the blinded bipolar signal was greater than the ME signal by 200% in only 6.6% of lesions. Attenuation of EGM signal during ablation was significantly greater and observed more consistently using ME signals (−71 ± 21% vs. −46 ± 36%, *p* = <.01). In the control group, attenuation of bipolar EGMs was significantly greater than the blinded bipolar EGM in the MiFi group (−57% vs. −45%, *p* < .001) which may reflect the bipolar signal being the one targeted in the control group. In EGMs recorded from sites where ablation lesions resulted in the termination of flutter or a bidirectional block (Figure 4), the mean reduction in ME signal was 85% (range 50–100%) compared to 37% (range 10%–80%) on bipolar EGM while in the control group, the mean reduction in bipolar signals for successful lesions was 50% (range 10%–86%).

There were no major complications in either group. One patient in the intervention group developed a groin hematoma which resolved with conservative management. One patient in the control group required permanent pacing for symptomatic sinus bradycardia following AFL termination. In two cases (one in each arm), bidirectional block of the CTI could not be achieved, most likely because of anatomical difficulties preventing adequate catheter contact with a critical part of the CTI. Both patients later developed clinical recurrence of AFL.

Two patients were excluded from analysis after enrolment, one because of atrial fibrillation on the table which could not be cardioverted, the other as the flutter was found not to be CTI dependent. In one patient, the MiFi catheter failed during the case and a replacement was not available; so, the final two lesions were delivered with a standard 8‐mm catheter, however, the patient remained in the MiFi group for final analysis.

## 4 DISCUSSION

This study demonstrates that the use of local ME signals alone enables ablation of the CTI, with the same acute procedural success as with bipolar signals from standard 8‐mm ablation catheters. There was no difference between groups in the primary endpoint of a number of ablation lesions required. There was also no difference in secondary endpoints of 12‐month recurrence rates, procedure time or fluoroscopy time. This is the first time, to our knowledge, these approaches to CTI ablation have been compared in a randomized, controlled fashion.

The amplitudes of the ME signals at ablation sites were higher than the amplitudes of bipolar signals obtained in the majority of cases. The greater ME amplitude did enable an interpretable signal to be identified in 13.6% of cases where it would have been uninterpretable using bipolar signals. Overall, the mean bipolar signal, which the operator was blinded to in the MiFi group, was similar between groups which raises some doubt about the benefit added by the ME signal. The finding of no overall difference in surrogates of effective ablation such as impedance drop suggests recurrences are not because of failure to visualize signal but to create an adequate transmural lesion.^10^

A critical component for adequate lesion formation is the ability to measure contact force (CF).^2^, ^11^, ^12^ A previous study performed with similar methodology suggested that CF, and particularly meeting a target of adequate CF and ablation time, the force‐time integral (FTI), was important in preventing recurrence.^2^ In that study there was a correlation between CF and impedance drop, implying greater tissue heating and lesion size, while in the present study there was no correlation with EGM parameters and impedance drop.^2^ Similar links between CF, impedance, and recurrence have been found in left atrial ablation for atrial fibrillation.^11^, ^13^ In contrast, a recent randomized study comparing CF‐guided to standard ablation did not find any difference in recurrence of CTI conduction (24%) at 3 months.^14^ This is somewhat surprising, however, the targets used may have been insufficient and/or not met in all lesions. The ideal target CF parameters in AFL are not known although we have previously suggested a force‐time integral (FTI) of 800 ± 10 gs.^2^ Without CF readings, the targeting of sites with high amplitude signals is often used as a marker for good tissue contact but the heterogeneity of values in the present study did not allow for a threshold value to be derived.^16^ Newer catheters combining the increased sensing of ME signals with the ability to judge contact force may inform this area further.^15^

Other investigators using non‐randomized methodology have reported similar findings to the present study. In a retrospective comparison of 50 patients undergoing AFL ablation with either a standard 8‐mm catheter (*n* = 37) or the MiFi catheter (*n* = 13), there was no difference in acute success rates or procedure times.^16^ In the same study, ME signals were thought to add diagnostic value (signal present on ME but not on bipolar) in only 3 of 13 cases. Another study comparing the use of the MiFi catheter, an 8‐mm dumbbell‐shaped catheter, and a cryoablation catheter found it to be inferior in ability to achieve bidirectional block and suggested reduced power delivery modulated by its temperature control system, although we did not find this in the present study.^17^ The same group examined using ME signals with a 10‐mm‐tip catheter to guide ablation in a point‐by‐point fashion and did find a higher “first‐pass” success rate using ME signals with less RF applications but without change in overall procedure time.^18^

The findings of the current study demonstrate that enhanced visualization of EGMs along the CTI using ME does not translate into improved ablation outcomes. When considering why the ME signals were usually, but not always, greater than the bipolar signals, several factors should be considered. First, the largest ME signal was sought prior to ablation with the intention this would represent a “critical bundle.” Second, differences in catheter orientation and angle may result in better tissue contact at either tip (ME) or bipolar pair. Third, bipolar signal may be larger but further away from a critical site if tissue is edematous or fibrosed or if the catheter orientation does not overlay the ablation line (Figure 3). Lastly, the direction of the flutter (or paced) wavefront relative to the radial ME or linear bipolar electrodes (Figure 2) may result in measurement differences.

**FIGURE 5:**
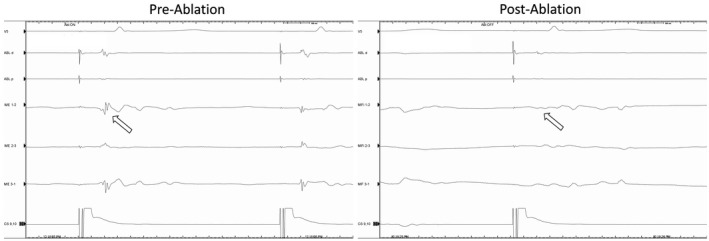
Signals seen with ME and bipolar electrodes in another patient before and after ablation achieving CTI block. ME amplitude is greater and while both signals diminish, the ME signal is almost completely absent post‐ablation. ABL, ablation bipolar; CS, coronary sinus; ME, mini electrode

**FIGURE 6:**
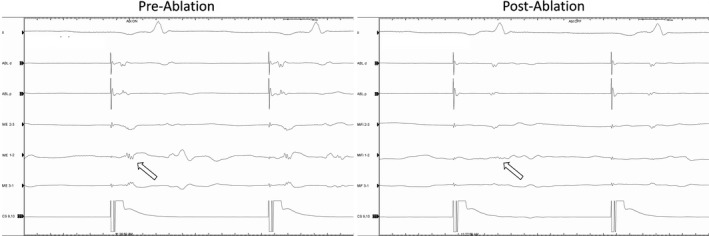
A further example in a different patient of signals before and after a successful ablation lesion with CTI block. ABL, ablation bipolar; CS, coronary sinus; ME, mini electrode

A larger and more rapid diminution of ME signals was observed compared to bipolar signals during ablation (Figures 5 and 6). However, animal models show that about an 80% reduction in ME signal is needed to be confident of a transmural lesion compared to a 50% drop in bipolar signal.^19^ As the signal reduction is easier to observe with ME, this might enable an approach where the ablation catheter is moved to a new position after an 80% reduction, although this remains to be evaluated. Equally, it has been pointed out that, in a horizontal configuration, the MiFi catheter would be positioned more proximally than a standard catheter using the largest signal, but this may paradoxically lead to less contact of the ablation tip with the targeted bundle and so less effective ablation.^20^

One of the purported uses for ME signals is gap finding across linear ablation lesions.^21^ While gap finding was not specifically investigated in the current study, the presence of superficial gaps found readily with ME signals might have been expected to result in reduced numbers of ablation lesions or procedure times in the ME group. No such differences were observed. Further, if a conduction gap is situated deep, below previously ablated or edematous tissue, then the absence of a “local” ME signal could be misleading. Another possibility to explain recurrence may be because of small channels of slow conduction not appreciated at the time of ablation and enhanced 3D mapping approaches may be needed to exclude this.

### 4.1 Limitations

Given the small sample size, it is possible an effect of small magnitude could be missed, however, there did not appear to be any numerical trend in the data in either direction and our findings are consistent with the previous observational studies.^16^, ^17^, ^18^ The recurrence rates of AFL observed in both arms of this study are higher than commonly quoted rates after CTI ablation but are consistent with many studies in the literature, suggesting recurrence rates may be a product of the intensity of follow‐up.^22^ Recurrences may be related to the specific methods of ablation used here and other methods such as the creation of a linear lesion by “pull‐back” along the CTI or the use of irrigated ablation catheters may have yielded different results. The use of two different catheter platforms may also have an influence on outcomes. For example, the MiFi catheter is less flexible than the standard 8‐mm tip non‐irrigated ablation catheter which may translate into less maneuverability and influence the ability to create effective lesions independent of signal sensing.

## 5 CONCLUSION

In this randomized controlled trial, the use of ME signals alone for CTI ablation, targeting areas of maximal voltage, was equally efficacious compared to a standard approach. There were no differences observed in the number of ablations required, procedure times or rates of recurrent AFL between the MiFi catheter and a standard 8‐mm tip non‐irrigated catheter. The use of ME signals allows improved visualization of EGMs compared to bipolar signals in a minority of cases. Prevention of AFL recurrence may be determined more by factors such as contact force that promote transmural lesion formation rather than improvements in signal sensing.

## CONFLICTS OF INTEREST

The authors have no conflicts of interest to declare.

## ETHICS APPROVAL

Obtained from the Review Board of the Metro South Human Research Ethics Committee.
